# Elements of Psychology
*Elements of Psychology. Part I. By J. D. Morell, A.M. London, 1853, Pickering.


**Published:** 1853-07-01

**Authors:** 


THE JOURNAL

OF

PSYCHOLOGICAL MEDICINE

AND

MENTAL PATHOLOGY.

JULY 1, 1853.

### Art. I.—ELEMENTS OF PSYCHOLOGY:*

Nothing, we believe, would better demonstrate to our readers the progress made in Mental Philosophy than a perusal of this volume. Mr. Morell is already well known by his "History of Philosophy in the Nineteenth Century," "The Philosophical Tendencies of the Age," and other publications of a similar character. The work before us may therefore be considered as the *result* of his studies, and as such merits particular attention, because it may be considered as expressing the point at which a cultivated and accomplished mind would arrive after careful research and prolonged and deep thought. The work is also eminently of a practical and readable character, and has therefore merits of its own. It may be also considered as a system of philosophy differing in many essential points from previous systems. We therefore propose to give our readers a short, but comprehensive, critical analysis of the work.

There are few men who will not recognise the fact, that in the acquisition of knowledge, upon any given subject, the mind is progressive. It is simply a truism. Now what is true of the individual is true of the mass. Hence, knowledge, *quoad*, nations, and the human race itself, is progressive. Again, few men will have failed to observe that the faculties by which the mind acquires knowledge, are not developed perfectly at once, but progressively. The man in his prime feels how much superior, in this respect, he is to commencing manhood;

* Elements of Psychology. Part I. By J. D. Morell, A.M. London, 1853, Pickering.

commencing manhood feels the working of faculties within him, unknown to his youth; youth looks down patronizingly upon childhood; and childhood enacts the attentive parent to helpless infancy. This progressive evolution of the faculties is a fact, in reference to nations as well as to individuals. It is a general fact, and as a general fact is adopted by Mr. Morell as the guide of his researches, and the basis of his philosophy. But important deductions flow from it. Mind works through a material organ. That material organ is presented to the neurologist in different stages of evolution and development, and working under different conditions, as to external relation, and as to the mind itself. The ultimate essence of recent researches shows that by virtue of some (as yet unexplained) mental operations, various machines are evolved in the organism fitted to the wants and destiny of the organism and regulated by this material organ; itself moved to act by mind in conformity with a pre-arranged and pre-ordered plan, dependent upon the Deity, acting as a Divine Providence. This plan is perfect in all its parts, and includes therefore the Universe. Hence, the mind works in relation to, or in harmony with, the entire scheme in creation; consequently the origin of its ideas lies in this primary harmony of the soul with the universe. From this point Mr. Morell traces the ideas and powers of the human mind upward, so far as experience and observation are the guides. Thence he ascends by the light of reason to that culminating point at which philosophy recognises the Divine Mind as the beginning, the middle, the end of all things—to that "goal, not only of Providence, not only of redemption, but also of the no less Divine laws of reason itself—that God should be ALL IN ALL."

To illustrate this programme. In the earliest periods to which the history of philosophy extends, we find the human mind principally engaged in theoretical or hypothetical researches; nevertheless, in the relations of number and space, a solid basis of science was found, and numerical and geometric proofs proffered results, which, by being removed from the possibility of scepticism, formed a fixed point for the mind amidst vain hypotheses and conflicting theories.

"This step once secured, it now became evident, that, having gained certain definite conclusions in these abstract spheres of thought, a platform was laid on which the reason could take its stand in order to penetrate into regions still beyond them. The results of geometry, after a time, assisted to bring unity into the vaster movements of the material universe, and gave the data on which science could build a perfect system of terrestrial and celestial mechanics. In like manner again, has our knowledge of the properties of bodies paved the way for a positive science of chemistry; while this, in its turn, enables us in due time to enter into the more complicated regions of life and organization. Lastly, physiology, when once pursued with all the aids of chemical analysis, tracks its way slowly upwards, through the vegetable and animal kingdoms, infusing law and order into the complicated phenomena which meet us in every step, until, at last, it touches, in its highest efforts, upon the region of *mind* itself. Here a new set of facts appear, which have to be conquered, reduced, calculated, and expounded, just on the same principles of scientific research as all the rest which preceded it; so that while every successive step in the evolution of the science, is based upon a fresh and peculiar region of actual facts, the course of science itself, the law of reason it follows, and the principles of investigation it has to employ, remain the very same throughout the whole series."—Preface, pp. vii, viii.

Such is the large and comprehensive scheme of inductive philosophy which Mr. Morell proposes to apply to psychology. That it fascinates by its simplicity, as well as by its comprehensiveness, cannot be denied. There is a simple grandeur in the idea that the culminating point of human reason should be the apex of a pyramid, the material and successive courses of which are derived from the entire cycle of knowledge. To the geometrician, the naturalist, the physiologist, it is a thing full of interest to find that mental philosophy need no longer be a science apart from his favourite studies. To the theologian it is of the utmost importance to learn that revealed and philosophic truth need not differ. To the religious man it is a blessed thought to

———"Find tongues in trees, books in the running brooks,
Sermons in stones, and God in everything."

Mr. Morell first defines his subject. Psychology (he states) is the science which investigates the essential properties of the human mind; those essential properties being all the facts and phenomena which belong to the mind of man *as such*, and without which it could not be considered perfect or entire. He distinguishes it from *Anthropology* on the one hand, which deals with the mental relations of man ethnologically and physiologically, and from Metaphysic on the other, which considers the mind of man in its *objective* relations. Psychology occupies itself simply with states, operations, and laws of mind; or, in other words, with the phenomena of consciousness.

A brief but very lucid "historical sketch" is next given. In this, Mr. Morell rapidly states and compares the various systems of philosophy which have engaged the attention of mankind from the earliest dates to the most recent. The sketch is critical as well as historical, and is followed by a section headed "Rise of a new method," which is a critical analysis of the various German systems of psychology. The following extract will afford a good idea of Mr. Morell's style and method:—

"Mental philosophy originally grew out of natural philosophy, and in every succeeding age the one has uniformly followed the track of the other. This is a fact by no means to be wondered at. The science of nature has always been the great pioneer to all the other sciences. It is *there* that the true spirit of philosophic investigation is fostered,—there that the most distinctive methods are elaborated,—there that the most definite results have hitherto been procured. So long as *natural* philosophy employed itself mainly with abstract ideas and plausible theories, *mental* philosophy did the same. When the former turned, on the contrary, to the work of observation and induction, the latter began to pursue a similar course. A like effect has flowed from the *actual results* as well as the *methods* of natural science. Each new discovery, which pours fresh light on the constitution of the universe, throws every other department of thought into new relations. Psychology, ethics, theology, alike feel to their very centre the mighty vibrations of every great truth to which the human intellect by the means of natural science is constrained to bow. It is no matter of astonishment, therefore, that the science of mind should ever become more or less modified by the relation it assumes to that of nature." —p. 21.

Now, these two sciences have assumed three different and fundamental relations to each other. The first is that of *absolute identity:* it is that form of materialism in which mind is regarded simply as the name we give to the functions of the brain and nerves. The second is that of absolute severance, or isolation from each other. As to the third, we quote Mr. Morell:—

"The third relationship which has been affirmed between the two sciences is based upon a deeper and more penetrating view of science itself; a view which includes both regions of research under one higher and broader unity. The science of nature, according to this third principle, is not *merely* a science of facts. Facts, indeed, must be diligently observed and classified, but then they must be rationally *interpreted;* that is, the reason of man must bring all outward facts and laws within its own sphere; must see their meaning, their purpose, their hidden analogies, their perfect unity in the whole scheme of existence. Viewed in this light, nature again becomes indissolubly linked with mind. The laws of reason are seen to pervade both alike, to bear the impress of the same creative mind, to be developed by virtue of the same great principles of universal existence, to conspire for the same ultimate purposes, and thus to form one harmonious universe."—p. 22.

The first distinct exposition of these principles was given by Schelling. The universe, according to his views, exists in three different stages or conditions; that of matter answering to the mechanical sphere of investigation, that of force answering to the dynamical, and that of organization corresponding to the rational. The same mind, intelligence, power, and purpose was alike through the whole; so that, from the lowest to the highest regions of existence, there is a steady development of life and being, in which the ideas of the Creator are ever more and more perfectly expressed. A great deal of Schelling's views were more imaginative than philosophical, but he gave life to a fundamental idea, rich in the grandest results. Christian Weiss took up this principle of organic development, and attempted to found upon it a complete system of psychology. He was followed by Heinrich Steffens, "a man of extraordinary versatility as well as uncommon compass of mind," and by Hegel. Beneke, of Berlin, adopted another system, which Mr. Morell designates as *empirical*. In this the predominant idea is connected with the working of the material organ, and the phenomena of consciousness are explained by the analogy of physical processes. Herbert, of Göttingen, who founded another school of psychology, employed the analogy of mathematical and mechanical ideas for the same purpose. The views of these and several other writers are critically considered, and then the following practical conclusions are drawn:—

"1. That the tendency of all speculations and researches down to the present time has been *to establish the entire unity of the soul as a real existence;* a doctrine which lies equally removed from the abstract view of the rational psychologists on the one hand, and from those who maintain an original multiplicity of independent faculties or impulses on the other. 2. That in pursuing the study of mind in its laws and operations, we must plant our footsteps primarily upon *human experience*. 3. That experience *alone*, however, will not satisfy the conditions of a *true science;* but that we must bring rational principles to bear upon the elucidation of the phenomena which experience presents. 4. That to study mind aright, we must not sever it either from the science of nature, or the science of *thought*, objectively considered. 5. That by such a union we combine the light which flows from empirical observation on the one side, with that which comes from speculation and reflection on the other. 6. That psychology is not a primary and independent science, and cannot be taken as the starting point of philosophy, universally considered; but that it holds its proper place in the logical co-ordination of the sciences at large, and will only be *perfected* when all the under-lying data shall have been duly explored and comprehended."—p. 37.

Psychology is thus then placed in a definite position in relation to experience and to knowledge. It is drawn fairly and irrevocably within the sphere of the inductive philosophy. This is done by breaking away all those barriers which false theories set up and thereby isolated the human mind from that great system of the universe, of which it forms, indeed, spiritually, a most essential portion. Henceforth, we must cross the gap which theorists have fixed between the unconscious and the self-conscious portions of the universe. Both are alike pervaded by the same great principles of reason and the same purposes of beneficence.

Proceeding in his inquiry with this fundamental idea as a guide, Mr. Morell notes some leading *analogies* relating to psychological research, and applies them. Thus his first observation of this kind is, that "The fundamental laws of reason alike pervade the mental and the material world." Here the term reason is used as including not merely self-conscious intelligence, but every process which we can recognise as springing from a well-defined effort to accomplish a given intelligible purpose. "For example, there is a geometry in nature as surely as there is in the human mind. The structure and the movements of the solar system exhibit on the one hand the most perfect agreement with mathematical laws, while the human mind, on the other, is so constituted that it cannot help recognising these principles of geometry and number as being absolutely and universally valid. What the one sees as *truth,* the other presents as *fact;* what the one knows *ideally,* the other embodies and exhibits as a *reality.* The laws of reason are alike existent in both; they lie equally at the basis of our ideal conception and of material realities. The physiologist recognises this automatic reason throughout all organization—in both animal and vegetable life. Here there is an infinity of the most exquisite adaptations and constructive contrivances wholly independent of any self-conscious intelligence, so far as human experience reaches, and only attributable by reason to the operation of the Divine mind. In these adaptations and constructions there is, however, not only the recognition of the truths of all sciences as *facts,* but the indestructible perceptions of beauty and of symmetry are simply the *ideal* counterparts of what *exists* in nature herself. Everything that is most striking in art, we know, is copied more or less from the forms of nature; and all deviations from such forms prove in the end to be contrary to our highest æsthetic sensibility and to the judgments of mankind at large." In reference to this point, we would direct Mr. Morell's attention to the researches of Mr. Hay, especially his latest work.* In that and in several works previously published the principles of geometry are applied to the determination of the beautiful in form and colour; and it is shown that harmonies of sound, of colour, and of outline are referrible to the same geometrical laws.

That interesting analogy we have just pointed out is also a striking illustration of a second observation made by Mr. Morell—"Science has discovered that a law of progression actually pervades the universe." The "law of progression" means this, that the works of nature present themselves to us in unbroken series, from the phenomena of bare matter to the highest products of organization and the vital forces. Here

* The Natural Principles of Beauty, as developed in the Human Figure. By D. R. Hay, F.R.S.E. Blackwood and Sons.

histology, comparative anatomy, and philosophical zoology illustrate psychology. In none of these is there a single fact without its bearing. The grand deduction from all the facts is this, that there is running through the series an ever-present, ever-active force, evolving an infinite variety of forms out of a common archetype. In Professor Owen's "Archetype and Homologies of the Vertebrate Skeleton," we have a valuable illustration. The bearing of this doctrine on embryology is obvious. Perhaps less so on instinct and on the generalizations of Lamarck and of writers like the author of "Vestiges of the Natural History of Creation." We will quote Mr. Morell :—

"Everything within its own limits is tending by virtue of a secret unconscious design towards an ideal, which may be perceived by the reason, even where not realized fully in fact. This is seen, for example, in individual organization. A flower shows the perpetual tendency to use all the advantages of its position to become the most perfect flower of its kind, on which fact depends the whole value of artificial cultivation. The animal frame appropriates instinctively all the means which lie in nature around it to become the most perfect animal. Circumstances may be wanting to admit of this result being reached, but the unconscious instinct is never wanting to strive after it."—p. 48.

Passing from individuals to species, to genera, and thence upwards, we find the same hidden reason operating progressively. Every species has an *ideal,* which constitutes its essential character, and which exists only as a secret power to reproduce its type. "The material exemplar is but a temporary manifestation, the ideal itself is an abiding reality ; one that existed before any individuals were produced, and that will outlive them all, as being a persistent law of nature, and consequently a thought flowing from its great author." So, also the genus only shows itself in producing a complete cycle of species according to a common exemplar or archetype, and in perfect numerical and morphic symmetry. Nature, *as a whole,* is but an evolution according to the same law. In the same way, Mr. Morell observes, as all the parts, in their several degrees of generalization, so, also, does nature *in her entireness* aim at an ideal perfection, which it requires an infinite number of steps and cycles to reach. Then there is a law of progression in each part, a law of progression in the whole ; and the only way to penetrate into the real secret of nature is to see the laws as laws of reason, at once *having* a purpose, and perpetually aiming at its fulfilment.

This law of progression, considered in its relation to psychology, presents the phenomena of the universe to us in various aspects. Mr. Morell's third "observation" is this, in reference to the law : "The whole universe may be conveniently classified into four ascending stages of existence, in each of which the laws of reason appear on a different scale, and operate in a different form :—these are, the inorganic—the merely organic—the sensitive—and self-conscious." As to the inorganic, the operating forces are chiefly *mechanical,* and may be calculated according to mathematical laws. There are other forces, however, (the correlatives of these) which link the inorganic to the organic, as caloric, the electric, magnetic, the chemical forces. They govern the universe *as a whole,* but are not embodied in an individual being—an organism. The point of division between the organic and the inorganic is not perceptible by science, but the *difference* is:—The organic powers or forces are correlative with distinct existences, each developed out of a primary germ, and exhibiting "in a real exemplar the ideal type after which they are formed." The sensitive arises from the organic; but as to the sensitive and self-conscious stages we give Mr. Morell's views in his own words :—

"Every ascending form of organic life, moreover, tends more and more to realize the one culminating purpose to which all the lower spheres of organization perpetually tend; that namely, of producing an *independent individual,* containing *in it* the power of self-regulation, and capable of reacting in opposition to the outward impulses of nature. This power of reaction accordingly, marks the commencement of what we have termed the *sensitive* sphere of creation."

"The brute is sensitive, but not self-conscious. Here, however, as everywhere else, we find an unbroken gradation; that is, we find a vast number of ascending steps, running through the whole animal creation, from bare sensibility on the one side, to self-consciousness on the other. First, the capacity of mere sensation becomes more acute; then the rudiments of other faculties begin to appear, such as memory—emotion—the power of adapting means to ends, and a number of animal impulses and affections. All these we put down loosely under the term *instinct;* but they evidently form a series of gradations, which, in their highest development, approach very near to the lowest type of humanity. They are stamped, too, with the same laws of universal reason as those which appear in man, only upon a lower stage, and without the accompaniment of self-consciousness."—p. 51.

Self-consciousness is most *manifest* in man; but just as there is a difficulty in drawing the line between the inorganic and the organic, and between the organic and the sensitive, so between the self-consciousness of man, and the higher forms of instinct in brute, there is to the curious inquirer into comparative psychology, an equally imperceptible line of demarcation. But nevertheless, while we cannot draw the distinction, we see both the *similarity* and the *difference.* Once within the sphere of human psychology, we see too, that there is the same order of ascending development from the lowest forms of humanity, ethnologically and mentally, to the perfection of human beauty, and of human intellect. Mr. Morell clenches these arguments as follows :—

"Thus, to sum up the burden of the whole remark, we see that each successive sphere in the universe of existence develops a new mode of life, which includes all that went before it, *with something more.* The organic sphere contains all the laws and phenomena of the inorganic. The sensitive world contains those both of the inorganic and organic, and the self-conscious, those of the inorganic, organic, and sensitive, *with something of its own beside.* The principles of reason, objectively considered, run through the whole; and the great law of progression accompanies each step, from the smallest atom of senseless matter, up to the most soaring spirit in the highest walks of human culture."—p. 52.

These are *facts,*—general, it is true—but of mighty import; not easily grasped by the mind new to them and to their relations; not to be comprehended at all by him who is of the earth earthly, and looks not beyond the little material world in which he grovels daily. Widely different are they to the contemplative observer. Looking at man by the light thus thrown upon him, he cannot but address his grateful praises to the Father of the spirits of all flesh, and say with deep conviction, "Thou hast made him a little lower than the angels, Thou hast crowned him with glory and honour." Looking again at the world invisible to material sense, but visible by the light of reason, and tracing the same law of progression upwards, what can we see but rank above rank of self-conscious existences, each transcending the other, each with the endowments of the lower stages, but *with something more;* beings glorious in form, in intellect, in knowledge; cherubim and seraphim, knowing God and his universe, and rejoicing in that knowledge with a lucidity and gladness that eye hath not seen nor ear heard, neither hath it entered into the heart of man to conceive. It is a strange thought, that philosophy can thus render certain to the enlightened reason that which seems wholly in the domain of faith.

Man being, then, the highest terrestrial development of the self-conscious existence, and having "all the spheres of the lower existences concentrated in him, as the true microcosm, we may trace out this law of progression, as it manifests itself in the growth and development of the human individual." This is Mr. Morell's fourth "Observation." To illustrate it, he concentrates into a few short sentences, the physiological doctrines of development, glancing from the primordial cell, which has powers in common with the vegetable cell, to the various phenomena of an instinctive and reflex character, and thence to the intelligence as it is evolved through its various phases. There is in the intellectual phases of the infant, the child, the youth, the mature man, a

*continuation* of the same great law which pervades the universe at large. It follows, therefore, in the words of Mr. Morell's fifth observation, "that the law of progression, as seen in the phenomena of nature, and concentrated in the life of man, should give us a clue to the formation of a scientific scheme of what are usually termed the human faculties." Arrived at this point, Mr. Morell touches upon the domain of the organic, within which so many of the causes of insanity and error lie. We shall state his views in his own words.

"The whole tendency of our previous observations has been to show, that the development of the human mind must be brought more or less under the universal laws of organic growth. The mind, *we know by experience,* depends for the manifestation of all its activities upon a material organism, which grows up, like all others, from a central germ. Hence, if the mind partake truly of an organic character, though in a higher region, the laws which apply to the progress of organic life *generally,* ought, *mutatis mutandis,* to hold good within its own subjective sphere, and the functions of the one ought to throw light upon the several stages of the other. This, then, will give us some *direction* as to the mode in which our observations of mental phenomena ought to be conducted. In forming a true idea of any living object, it is not sufficient to analyse it into its component parts. We can form no conception of its true nature without taking into account its growth, without viewing its successive developments in relation to each other, without regarding it, in short, as the centre of a history, the issue and aim of which we must watch, as well as each of its separate stages. What idea should we form of the flower, if we saw it only in the leaf, or only in the blossom, or only in the fruit? * * * Such objects do not exist by mere agglutination of particles *ab extra;* they come by a growth, which springs from one central point, and then retains its perfect unity of idea and purpose through every succeeding phase of its existence."

"Applying this analogy then, to the human mind, we are led insensibly, and yet inevitably to view it, not as a mere combination of powers and faculties, but as one undivided power—a *spiritual organism,* if we may so term it—which throws out its energy in many directions, evolves a vast variety of different activities, and passes through a whole series of ascending stages, without losing, even for a moment, the unity either of its nature or of its highest purpose. To have a psychology that leaves life, growth, organic unity, and progressive development out of account, would be the same as to have a physiology based upon the mere anatomy of the frame, the whole phenomena of life being disregarded or disowned."—(p. 57.)

Now the law of progression is none other than a series of successive phenomena of antecedents and consequents linked to each other for a definite end and an unchanging purpose. That end and purpose is perfection—the consummation of that which is designed. Applying this doctrine to psychology, Mr. Morell asks what is the *ideal* of the human mind—the thing designed—the end proposed by its successive evolutions?

"According to this view the ideal aim of man's nature must be to elevate himself above all inferior determining influences *ab extra;* to gain complete freedom of action; to present in himself the most perfectly self-conscious, and the most perfectly independent manifestation of intelligence and will, in their highest and purest sense. It is only in rising to this elevation that he can lay the topstone upon the vast edifice, which the whole effort of nature is endeavouring, in all its progressive developments, finally to construct. Very much is necessary to contribute to this end. No man, for example, can be *free* without knowledge; for freedom itself, deprived of the light of reason, were but a blind impulse. No man again, can be truly free, or rational either, without *right affections;* for, with *base* affections, he is a slave to the lower purposes of existence, instead of a living manifestation of its highest ideal."—p. 58.

These considerations lead to the clue to a correct classification of the mental faculties. The law of progression becomes the law of *evolution.* The means to the end are gradually *evolved*—the faculties appear *pari passu,* as the great object becomes more apparent.

"If, then, we have designated accurately the true ideal of the human mind, *we hold the two ends of the whole chain of phenomena, between which all the development of its powers must necessarily lie.* Man is, at first, a mere creature of sensation and instinct; from that he rises to the power of perception, separating the world from himself, and becoming conscious, *here* of his own identity, *there* of the universe around him. After this, he attains to the power of representation and expression, stamps upon objects their distinctive names, classifies and generalizes them, and penetrates them with the light of the understanding. After this process of *analysis,* begins the higher process of *synthesis.* The objects separated and classified, are now reconstructed in scientific order, and the truths which were first seen only by the light of sense and intuition, are now comprehended by the clearer light of *reason.* With the development of the reason are given the conditions of the development of the *will,* which rises through like gradations, from mere instinct to conscious self-action, and at last to the height of perfect freedom."—p. 59.

Thus there is in man's nature, as in all creation, a progressive evolution towards a pre-ordained perfection. The perfection of the intellectual portion of his nature is *reason* in its most explicit and philosophic form,—"reason which penetrates into the principles of truth, and grasps the whole sum of knowledge in its entireness and its unity." The perfection of the emotional portion is *love,*—"love to every thing good and great,—love to all that draws us towards the highest and purest state of mental existence." The perfection of the voluntary powers is freedom—"a freedom that is antagonistic to all lower and material influences, and which is bounded only by the co-ordinate promptings of perfect reason and perfect love." These are the three great modes of mental manifestation, and in accordance with the fundamental principles of Mr. Morell's system, psychology commences with the study of these modes in their lowest and most restricted sphere of operation. Every higher manifestation contains the elements of the mode of mental action below it, with something more; the three being evolved upwards consentaneously and in parallelism. The subjoined shows Mr. Morell's fundamental arrangement:—

MIND.

*As*

| | I. INTELLIGENCE. | II. FEELING. | III. WILL. |
|---|---|---|---|
| First Stage. | Sensation. | Pleasure and Pain. | Practical Instinct. |
| Second Stage. | Intuition. | Sentiments. | Passions. |
| Third Stage. | Representation. | Affections. | Art. |
| Fourth Stage. | Thought. | Love. | Freedom. |

Such is the scheme of investigation. Previously, however, to entering into details, Mr. Morell devotes a chapter to "the Genesis of Mind, and its Connexion with the Body," feeling that the question "how is the mind produced?" arises at the very threshold of inquiry. He submits the inquiry to the searchings of *experience*, the only sure guide, and looks at the facts and analogies of the question. All the results of the best and most recent physiological researches point to the doctrine of origin from a single cell as that which will endure. The embryo man is no exception from the general law; each individual human being when first recognisable as a material fact, exists simply in the form of a microscopic cell. But the material form is nothing; what the cell contains potentially infinitely transcends the mere matter in importance. That (to put the question in its simplest form) is the power of *self-evolution* both as to mind and matter; and evolution most exactly progressive and constantly tending to that perfection which we have indicated. We need not state the facts of development now well known to every intelligent and thinking man; we will only state the proposition which Mr. Morell bases upon these facts, namely, that the production of that cell and the presence of that power of self-evolution, "cannot be conceived of except as resulting from a previous type, that is, from a THOUGHT or plan in the creative mind, which was designed to realize itself in a material form." It follows, therefore, that we cannot imagine the *real* to exist in man, without imagining also the pre-existence of the *ideal.* If the germ be material it is also the dwelling of the spiritual; if it contain potentially the entire development of the *body,* so also there is potentially present the *spiritual* part—the entire development of the soul. It is the two in union which constitute the *individual;* divide them—that is, destroy the material arrangement—and you annihilate that manifestation, at least, of the spiritual part. Mr. Morell is evidently a Stahlian in doctrine, but adapting that doctrine to modern research. With him the mind is no other than the same force on which the adapting operations of the "vital principle" depend. This doctrine of the unity in origin and effects of the mental and "vital principle" is the key to Mr. Morell's philosophy. He rejects the doctrine of the materialists on the one hand, who maintain that mind is merely a function, and on the other the doctrine of the Dualists, who assert that the soul is an essence physically separate, and separate from the body. We will subjoin Mr. Morell's argument on this point:—

"What, in truth, is the body taken alone? Simply a corpse. There is no unity in its constitution. It is a compound or accretion of particles, which, left to themselves, dissolve with the utmost rapidity. Without life, moreover, there is no unity in its *design* and *purpose.* One part does not work with another; it has no mechanical adaptation to any given end,—no use to subserve in the creation around it. Add the principle of life and intelligence, and the whole becomes *one*—one in its conception, one in its purpose, and one in its entire nature. But what objection, it might be said, can be urged to the view, that the soul is a spiritual *substance,* distinct from the body, and superadded to it? The objection is this—that every conception we can possibly form of such entity is purely *negative.* Of spirit, substantively considered, and apart from a material organization, we have no experience, and consequently no positive idea. The only method in which it can be defined as a substance is, by taking the realities of which we *have* experience, and abstracting one property after another, until we have an entity *without* extension, *without* resistance, *without* parts, *without* divisibility, &c. &c. After such a process of abstraction, that which remains is a mere *negation*—a remnant to which we can reasonably assign none of the concrete properties of life and activity."—p. 74.

Mr. Morell supports his views by various facts from physiology, and especially that department of the science which treats of the functions of the nervous system. Those of central reflex action, and especially of "unconscious cerebration," derive light from statements like the following. In reference to the perfect coincidence of the soul and the body, Mr. Morell remarks:—

"The reason why this has not been more clearly perceived, is chiefly owing to the pertinacity with which the human soul has been confounded with the human *consciousness.* The soul, as we have shown, is *prior* to consciousness. It exists *unconsciously* from the formation of the first cell-germ; it operates *unconsciously* throughout all the early processes of life; it acts unconsciously even in the greater part of the efforts which subserve our intellectual development. All the most complete researches into the nervous system confirm this view of the case. Nervous force and mental force are perpetually interchanged and interchangeable. Sensations, ideas, feelings, affections, purposes—all play backwards and forwards between soul and body with the most perfect interpenetration. The soul is in the whole body, in every part, in every nerve; it forms the peculiar essence of humanity, and with the body it constitutes the reality and the unity of the individual man. Of physiological writers, Unzer has exhibited this unity in the most striking way, and by the vastest array of actual facts."—p. 75.

Mr. Morell supports his position by critical notices of philosophical systems as well as statements of fact. This absolute identification of the soul with the body seems at first sight so thoroughly materialistic, that some caution is required in advancing it and stating the grounds of it. An intuition of a future generally, and of future life for man specially, is part of human nature—perhaps the most precious gift of God to man. Rightly, therefore, Mr. Morell inquires how in presence of his doctrine we can conserve the "great moral truth" of the immortality of the soul. This is his explanation:—

"Were the real regarded as prior in nature and development to the ideal, so that the soul merely appeared *phenomenally* as the result and function of the bodily organization, then, indeed, the hope of immortality could have no foundation in our psychological principles. It is, however, indispensable to the whole theory we have propounded, that the ideal shall have assigned to it a *prior* and an independent existence; that it should *constitute* the individuality of the man by its union with a bodily organization; and, finally, that it should comprehend in itself the essential conditions of one continued existence, throughout all the changes to which our bodily organization is exposed."

"If this be the fact, then the only thing which passes away with the dissolution of the body is the *mundane* individuality, *i. e.,* the entire complex of physical causes, on which the peculiarities of our mere *human* life and temperament depend. The very analogy, however, of a mundane birth, suggests a still higher birth, viz., the entrance of the pre-existent and immortal *ideal,* as trained and developed by human life into new relations; its connexion with a superior organization; and its advancement to a higher and purer individuality. In this view, death is but a crisis in our being, the dissolution of the *earthly* tabernacle,—'not that we may be unclothed, but clothed upon, with that which is from above.'"—p. 83.

That spiritual element which existed before the body was formed, will exist after that body is destroyed or disintegrated. This is the conclusion of common sense. *How* it existed before, and *how* it will exist after, are circumstances beyond the reach of human research; we need not, therefore, inquire into them. It is a pleasing feeling, nevertheless, to find our *faith* in a future life thus strengthened alike by the phenomena of nature and the conclusions of philosophy.

Passing from these general doctrines, and the region of speculation on which they border, Mr. Morell proceeds with the more immediate object of this volume, namely, *the analysis of the intellectual powers.* We have already, in the table previously given, indicated the order of precedence which Mr. Morell has laid down for the development of his system of psychology. Under the head "Intelligence," in that table, the reader will observe the four "stages," or divisions of the intellectual power which Mr. Morell considers. In Chapter 3, *sensation* is considered in all its forms to the rise of self-consciousness. In Chapter 4, perception is analysed as *intuitive* intelligence. In Chapter 5, memory, imagination, and association, and the *sematic* power (representation by *signs*, a language) are considered as collectively constituting "intelligence as representation." In Chapter 6, the understanding, abstraction and generalization, the reason and its appliances to observation, experiment and speculation are considered, these constituting intelligence as thought. The 7th and last Chapter is a summary of modes of verification, by facts drawn from various sources, and by philosophical considerations. We presume in the succeeding part or parts "Feeling" will be considered in its four stages, and then the Will in its relations to Life, Art, and Morals.

The first stage to be considered is *Sensation.* Mr. Morell defines intelligence as "including all the mental phenomena which contribute immediately to the production of knowledge." The phenomena of *sensation* are the earliest and most fundamental. Where sensation begins is to Mr. Morell, as to all other philosophical inquirers, an undiscovered point. There is a *stage,* however, of sensation in the development of the human being, which is definable enough. That stage in which the soul begins to act consciously for itself, but yet only responsively to the stimuli of physical influences, is the "purely sensational stage." This general fact brings us at once to the *nervous system* as the seat of respondence to physical influences, and Mr. Morell gives a brief but very effective summary of the anatomy and physiology of that system, in relation to sensation. First, the merely automatic acts are considered—Dr. M. Hall's excito-motor phenomena; then the sensori-motor phenomena (rising above these) are stated according to Dr. Carpenter's views; and finally the cerebral reflex phenomena, and their relations to intelligence in accordance with Dr. Laycock's doctrines. These have their respective seats.

"Each of the several centres to which we have already referred, may thus become a point from which impressions are reflected, and with a wholly different result in each case. Thus, if impressions are reflected from the spinal cord, muscular movements *alone* follow without any sensation or consciousness whatever. * * * Next, if the impressions are reflected from the sensory-ganglia, then feeling and consciousness will be actually awakened; but the movements will be wholly *automatic*, influenced simply by the sensation, and not at all by the will. Such movements, for example, as winking the eye, to prevent injury; shrinking to avoid danger; balancing the system to prevent falling, and numerous others come under this class; movements, which in the lower animals usually take the place of the *will*, and in man undertake the same duty, whenever the will would not decide quickly enough to accomplish the purpose required without physical inconvenience."

"But, thirdly, it has been shown by Dr. Laycock, that the cerebrum itself is also a centre of *reflex action*, that the nervous impression may excite some *special* activity there, and that both ideas and emotions may flow on from this excitement, without any of the governing power of the will. This is seen in dreaming, still more clearly in somnambulism, whether natural or artificially superinduced; and it not unfrequently forms the prominent characteristics of men, who possess large intellectual faculties and strong emotions, with no corresponding power of voluntary self-government. Indeed the brilliant qualities which appear in men of genius, often result from the spontaneous reflex action of the cerebrum, urging the individual onwards with extraordinary force in one particular train of thought and feeling, independent of any effort or even of any desire of his own. To a certain extent, indeed, the will may *guide*, it is never able actually to *originate* them."—p. 100.

Over all these centres, there is, finally, the dominant power of the will. This is the distinctive feature of humanity. Mr. Morell considers that the action of the will stands in *correlation* with that state of the nervous system which exists when it is brought into full and united action according to the laws of reflex action. In this opinion he finds himself in opposition to the phrenologist, and in advance of the physiologist. To do Mr. Morell full justice, we will state his ideas in his own words.

"It appears to me, however, viewing the question upon rational grounds, and following the analogy of the reflex acts generally, that, just as an act of the will embodies the effort of the whole man, implying, at the same time, intelligence, feeling, and force; so, physiologically speaking, this state of mind will stand in correlation with the *total affection* of the nervous system. Affect the spinal cord, and we have simply excito-motor actions; affect the sensory ganglia, and we have consensuous actions; affect the intellectual and emotional regions, and we have emotional and ideo-motor phenomena. Lastly, if the affection reaches its full height, and brings the whole nervous system into one united attitude of attention, then we shall have that state of purely voluntary activity which expresses the concentration of the whole man in the deed and effort of the moment."—p. 101.

Mr. Morell wishes it, therefore, to be understood, that the spiritual principle manifests will in a lower sphere in relation to sensation, in a higher sphere in relation to reason; in both there is an effort of the spiritual principle adapted to the precise circumstances of the case. In support of these views Mr. Morell gives a diagram, in which the relations of the "intellectual and emotive phenomena" to the nerve-centres are indicated. The anatomical and physiological views therein indicated are as yet quite hypothetical; all we can state positively is, that there is an *epicyclical* reflexion of impressions as we ascend upwards in the scale of development, and a consequent or coincident wider sphere of external relations, until the whole nervous system is brought into action by an impression, and the will operates thereon. This action of the will is downward, as Mr. Morell remarks, following the hypothetical track of the impression upwards. We can regulate the thoughts and emotions by the will, but we cannot perform voluntarily those series of movements which depend upon the excito-motor or sensori-motor centres. Nevertheless, the *ideas* which arise in the same region as the will reach downwards upon the sensory apparatus, and produce the effect of sensory impressions *ab externo.* To this class of phenomena belong the mesmeric and electro-biological manifestations of aberrant mind.

Mr. Morell passes from sensation in its physiological relations to its psychological connexions. It may be described "as resulting from the attention which the mind gives to the affections of its own organism." "The entire mental process which is necessary to produce sensation consists, according to what we have shown, in the mind receiving the affections of the body, and then embodying its own affections." Sensation takes place at the moment of action and re-action in the nerve-centre. Although Mr. Morell gives accurately enough the views which have been adopted by most thinkers, from Unzer downwards, we do not think he has penetrated the mystery in relation to pleasure and pain. On this point, however, he may have more to say in the next part, in which these states of the consciousness will demand predominant consideration. We will therefore defer criticism until those views are promulgated. We think the order and nature of sensation generally is well shown in the following passage.

"The nerves may be subjected to many impulses, affecting the body, and indirectly the mind, without those impulses ever coming into consciousness; and conversely, many actions may go forth from the vital forces (urged and impelled as they are by the soul itself) with an equal unconsciousness of their very existence. The instant, however, the whole circle comes into operation (like a magnetic chain), the instant an affection reaches the centre, provokes reaction, and is impelled back to the other pole, the light of consciousness at once breaks in, the mind is roused to a perception of what takes place within its own organic sphere, and a *mental* fact indispensable to all our future knowledge is the result. Sensation, accordingly, holds exactly the middle point in the soul's development between consciousness and unconsciousness. On the one side of it are processes which are termed *vital;* on the other, processes which are termed *spiritual; in* sensation itself the vital and the spiritual are indissolubly combined."—p. 109.

The senses are next treated of, individually, the views of Erdmann, Karl Schmidt, and Fischer, being mainly adopted by Mr. Morell, and differing, therefore, from those of the English schools. The rise of self-consciousness is coincident with the commencement of sensational life. The unity of sense is its primary form; all the impressional phenomena lead to a middle point, and there they become related to each other as parts of one continuous life. From this stage upwards, duality is present; on the one side is the abiding self, on the other, the world and its phenomena. "Self is first perceived as that which is *not phenomena,* the world is first perceived as that which is *not self.*" We shall not notice these views further at present, but pass on to the consideration of the next stage—namely, *Intelligence* or *Intuition.*

Mr. Morell first investigates briefly the theories of *perception* which have been advanced, and points out their faults and imperfections preparatory to setting forth his own doctrines, which, in this part of his subject, as in others, are eclectic. We shall not follow him in his critical analysis, but simply state, that according to his views our perceptions—"our immediate experiences of the world without are mental phenomena, which arise out of the direct conflict of mind and nature, resulting, therefore, neither from the mere operations of the one, nor the mere impressions of the other, but from a combined and harmonious action of both." To illustrate this somewhat vaguely general proposition, we will take one or two of Mr. Morell's examples:—First, as to the perceptions of heat and cold. It is clear that the affections which we experience when we touch a hot or cold body, are in us, and not in the body touched. There is a power in nature which stiffens, dissolves, expands, consumes; this power comes into contact with our organism; a "conflict" between the power and our organic condition (both mentally and bodily) arises, and it is out of this conflict that the perceptions of heat and cold arise. So it is also with the phenomena of touch and taste, and smell and sound.

"The particles or properties which affect the palate, or reach the olfactory nerve, are, apart from ourselves, mere chemical agencies, by which one force in nature acts upon another. Tastes and scents do not exist in them apart from the counter-operation of our own mental and bodily constitution. Take away the percipient mind, and all the enjoyments of the feast, all the fragrance of the flower, and the whole of the association which they embody, vanish as with a single and magic stroke. With sound the case stands precisely the same. Externally to ourselves there are movements and vibrations in the atmosphere, but there is no *sound* until those movements affect the living ear. The whole world of tone—the grandest harmony, the softest melody, the living voices of nature—all *exist not*, except as we co-operate, each one individually, in their production, nor can their characteristics be for a moment separated from the whole constitution of those who realize them. The perceptions of tone and harmony, indeed, we know very indefinitely, according to the temperament of different individuals, and therefore can have no common type or representation out of ourselves.—p. 131.

Hearing is the sense of *motion;* with an organ adapted to the harmonious movements of the universe, and a soul behind that organ capable of feeling the changes duly excited within that organ, to what heights of melody may it not ascend—to what exquisite harmonies may it not reach!

"* * * Look how the floor of heaven
Is thick inlaid with patines of bright gold;
There's not the smallest orb which thou behold'st,
But in his motion like an angel sings,
Still quiring to the young-eyed cherubim;
Such harmony is in immortal souls;
But while this muddy vesture of decay
Doth grossly close it in, we cannot hear it."†

Mr. Morell discusses *acquired* perceptions as well as the primary. He argues that, strictly speaking, *every* perception is acquired. The impressions which the mind receives at first cannot be properly interpreted. "Trace after trace has to be laid up; many of them to be compared together; the intimations of one sense to be used in correction or elucidation of another; and thus gradually the sign-language

† Merchant of Venice.

of sensation has to attain the *meaning* which we denote by the term *perception*."

The next step in Mr. Morell's system is to show the psychological identity between perception and intuition generally. What we are immediately conscious of in perception is the *qualities* of matter; these constitute to us the real elements of the material world. The appreciation of these qualities is as much an intellectual exercise of the mind as any other; the mind apprehends extension as readily as it apprehends beauty: under suitable conditions harmony is appreciated just as directly as time or space. The real object of intuition is no more material in the one case than in the other. So if the elements of knowledge, which come to us through perception and intuition, be traced to their higher intellectual forms, we find that while the rational laws of harmony, beauty, moral science, and natural theology, are based on our higher intuitions, the abstract truths of mathematics and physics are based upon our perceptions. To meet the argument which might be raised from *comparative* psychology, Mr. Morell notes a wide and fundamental difference between the perceptions of the human being and the brute. The brute acts towards objects perceived by it in reference only to its instincts; a conscious separation is instantly effected by the human faculty between the subject and the object.

"The animal does not think within itself I am a dog, or a horse, and that is a hare, or a corn-field; it is simply impelled *by the force of instinct* towards the object, without any apprehension of its own personality as distinct from the thing presented to it. On the other hand, the child or the savage, without the least culture whatever, *consciously* separates self from the objective world in the very first abstract act of *perception;* and it is exactly here, in this very act, that the intellectual quality of perception is first manifested. In the separation of subject and object, all thought is primarily cradled; and wherever that distinction takes place, everything else peculiar to the human intellect is able to follow."—p. 141.

In perception proper the mind is, therefore, in a state one degree raised above sensation: it is self-consciousness first acting for itself. But with that intellectual state of mind there is also a feeling of the pleasing in the objects perceived and the converse. The young mind drinks in all possible kinds of impressions long before it comes to the use of words, or has received instruction. It gazes with an undefined sense of wonder and admiration at the beauty which surrounds it on every side. Hence these feelings (the basis of all æsthetics) are as intuitive as perception itself.

"We contemplate an exquisite flower, or a summer's landscape, or the starry heavens,—and what do we there PERCEIVE? not merely phsyical qualities—not merely shape, size, colour; we perceive *far more* than this. An indefinable *sense of beauty* steals over the soul, which, as a mental phenomenon, is too real to be denied, and which we find, on reflection, to involve the dim realization of some of the deepest thoughts and realities of existence. The fact that the same amount of capacity does not exist in every individual for appreciating form and beauty, is in no respect contrary to their intuitive character. All perception and intuition, as we before showed, is really *acquired* by a spontaneous mental process, acquired by some, too, far more readily and rapidly than by others. In Homer, Raphael, Shakspeare, Goethe, how wondrous were the glimpses opened by this inward faculty; how true the ideas which the outward world reflected into their inmost soul! Such instances, however rare, yet exhibit to us in a magnified form the reality of the intuitive powers, as regards the appreciation of order and beauty."—p. 144.

The same views apply to all our sentiments. In *harmony* we have the same order of facts; in the moral sentiments, in the intuitive apprehension of right or wrong, or conscience. "Too often, indeed," Mr. Morell remarks, in one of those pithy sentences fraught with deep wisdom, with which his work abounds,—"Too often, indeed, the self-conscious and reflective knowledge of good and evil implies the *loss* of inward innocence, and the tarnishing of the moral nature by sin." Having eaten of the fruit of the tree of knowledge man discovers his nakedness. So again in the highest of all intuitions—the religious—Mr. Morell traces the same law. God has implanted them in the very soul of man. They may be directed and expanded by theoretic ideas, but they cannot be *created*.

"This would be to reverse the whole order of man's mental development, and stand, at the same time, in plain contradiction to that uniform body of experience, which shows religious *life* to be at once the forerunner and the necessary condition of an articulate faith. The realization of the Infinite—the Divine—the Holy and Perfect *One*, in the depths of our self-consciousness (*i. e.*, in the religion of experience) is prior to all *theory;* and, when attained, is a wholly different thing from the view we take of God intellectually in a theological system. In the latter, we see simply the understanding busy with a series of abstract ideas; in the former, we have a realization of the Infinite in the natural and truthful mirror of the religious feelings."—p. 147.

Mr. Morell next proceeds to discuss the essential characteristics of our intuitive intelligence, and then devotes a section to the phraseology employed. Our shortening limits warn us that there are yet two important stages of the human intellect to traverse; we therefore omit these without comment, and pass on to the—

Third stage of intelligence—*Intelligence as representation.* No intuition can be definite so long as it remains wholly and solely an intuition. Intuitive knowledge is less an object of thought than of feeling; to be subjected to the process of thought it must be projected out of the mind, and represented as an independent intellectual reality. The mind must place its intuitions before itself. This done, they become *representative ideas.*

*Memory.*—This is the first phenomenon to be considered; it may be traced, like all other mental operations, from the earliest trace of sensation. All our sensations are more or less capable of *reproduction.* Every impression to which the mind gives a response leaves, in some unknown mode, an inner trace behind which is permanent. This is the primary and instinctive form of memory. When *intuitions* are revived, so that we are fully conscious of the affinity between the type and the antitype, that is memory in its proper meaning. The process of recalling *ideas fully formed* and expressed in language, is *recollection.*

*Attention* arises with memory. The mind is instinctively dissatisfied with the vagueness of the knowledge supplied by intuition. We desire to scan individual objects more closely; we endeavour to grasp the intuition with the utmost degree of clearness; to fix it; to stamp it upon the soul. This act is the act of attention. During this process the mind seizes upon the leading features in each perception, brings them prominently forward, and thus *creates* by its own inherent power a new aspect of the entire phenomenon. After this, although the object may pass away, the idea of it, so constructed, will be capable of almost perfect reviviscence.

> "Memory, accordingly, is nothing more than the REPETITION, apart from the real phenomenon, of the same process of attention which the mind has already performed in its presence. If it be originally performed with great intensity, and under the stimulus of strong feeling, or if it have been repeated a great number of times, the reproduction will be so much the easier. The reason why such reproduction can take place *at all* is, because the process of attention, which necessarily precedes memory, is an act of the mind's own intellectual power, and any act which it can do once under the stimulus of the real object, it finds little difficulty in repeating, even when that object is no longer present."—p. 170.

*Imagination* is the next step in the mental growth. The image or idea we form by attention and memory may become so wholly mental or ideal, that after a time it is retained in the mind as a fixed representation or idea, independently of its connexion with any given object or event in nature; and without reference to time or place. The representative faculty thus acting, is termed imagination and it is manifested under two forms, one higher than the other. The lower is that just described, and is *reproductive* imagination; the higher is the

*productive* or *creative.* The lower stores the mind with ideal images, constructed out of our immediate perceptions, constituting *types* with which we can *compare* any new phenomena. Did they not exist, every fresh perception would be a new wonder; and we could not benefit by experience. These states of mind are respectively indicated in a child or a savage, and an adult or civilized man, looking at a work of art, or a natural curiosity, for the first time; the child or savage is all wonder and curiosity; the adult or civilized man mentally compares it with some idea already stored in the mind. The productive or creative imagination works upon the materials thus stored up by the reproductive. It is a second stage removed from the real. The reproductive imagination has stored in the mind the idea of a diamond and the idea of a rock. The productive imagination combines these, and forms out of them a purely mental creation—namely, the idea of a diamond rock. The creation of these ideas is closely connected with the *succession* of ideas through the mind, and with what has been termed—

*The laws of association of ideas.*—Consciousness itself is only possible under the condition of a succession of phenomena in the mind. As to perception, this succession is necessitated by the changing influences of things around us, but when the world is shut out, and the mind is occupied wholly with its own creations, there must manifestly be an order of succession according to some inward laws. These laws are placed by Mr. Morell in four categories. The first is that by which successive ideas are associated, in virtue of their essential affinity, when one idea necessarily suggests another. Thus, father suggests son; light, darkness, &c. This is the law of *correlation,* the ideas being correlated to each other; it may be reckoned absolute and universal. The second is the law of *similarity;* thus the arm of a man may suggest the arm of a chair. This law of association of ideas is the basis of analogy; it also enables us to carry on the processes of abstraction and generalization, by directing us to similarities, and ministers therefore largely to scientific research. It is also of great importance to the creative imagination. The third law is the law of contiguity, and the fourth the law of individuality or idiosyncrasy, which seems rather an accident of the other, than a special development. It is founded upon the fact, that every individual has a special mode of mind. It is the law of habit, and of hereditary conformation. Mr. Morell dismisses this law much too briefly, and is clearly in the dark as to its importance and extent. It bears directly upon the doctrine of innate ideas, and upon the acquisition and transmission of instincts, habits, and mental constitution, in relation to bodily organization. The doctrine of psychical *sub-strata,* sketched and partly developed by Dr. Laycock, in his "Essay on the Reflex Function of the Brain," is worthy Mr.

Morell's consideration; it is this which Dr. Carpenter alludes to in the extract from that writer's "Principles of Physiology," given by Mr. Morell, in a foot-note. It will not be possible, indeed, to develop any natural system of psychology without a full inquiry into the relations which ideas bear to that congenital and histological structure of the nervous system, upon which all innate modes of thought and adaptive action depend, and which, in reference to mental and quasi-mental operations, Dr. Laycock has considered in the paper referred to.

*Signs of ideas. The sematic power.*—The next step in the stage of intelligence is to make the ideas distinct *objects* of perception, so that the *inward* representation to the mind may be made external, able to be recalled and dismissed at pleasure. This is done by the use of signs, or, in other words, by language. Symbolic language was, and always is, the first stage in this objective representation of ideas. It is not *necessary*, however, that the symbol should have any affinity in *nature* (as it always has *primarily*) with the thing signified. With a very slight increase of intellectual power, an *arbitrary* sign will answer the purpose equally well, and more conveniently.

"One step more is only necessary to make the sematic process complete—namely, that instead of using some sign, existing apart from ourselves, for the embodiment of the idea, the mind should construct freely for itself the *idea* and the *sign* likewise. This is actually accomplished in the very first articulate word that is uttered; so that here, at length, *in the word*, we see the triumph of the representative faculty. In the construction of the elements of language, it has raised itself above feeling, above intuition, above all the inward images of imagination; it has created a *new external world*, transferred into that world the phenomena of its inner life, and achieved the first step *in the freedom of human thought*."—p. 88.

The views of Mr. Morell as to the origin and uses of speech are singularly interesting and instructive:—"We *perceive* the phenomena of nature by virtue of the adaptation existing between it and our own minds; we *comprehend* them only in one form of language. *The world must be known through the word.*" In sentences full of meaning like these, Mr. Morell develops his views, for which we must refer to the book itself, as any analysis we could give would only show them imperfectly.

*Intelligence as Thought* is the fourth and last stage of its development. We observed that the recal of ideas expressed in language is *recollection*. This particular form of memory introduces the child to a world of ideas already formed and expressed, and with this world thought is more particularly, yet not exclusively engaged. The path to *general* knowledge is now open; the mind is no longer restricted to the outward material object, but acquires *general* ideas already perfected by the laws of association." Nevertheless, its *mode* of action does not differ in the higher stage; it *compares* the terms which express ideas so as to estimate their relative agreement and disagreement, and passes various kinds of judgments upon them. An abstraction may be compared with an abstraction, or a generalization with a generalization, or the latter with the former. The mind then expresses the result of these comparisons (which is a complete thought) in a sentence or proposition. "The mental activity by which we compare terms, find out their exact agreement or disagreement, give expression to this in propositions, and deduce other propositions from them, is that which, *par excellence,* bears the title of THE UNDERSTANDING."

The *reason* is the culminating point in the development of the intelligence. Its function is to create *knowledge;* knowledge at once real and universal, applicable to every fact and every phenomenon. Its product is *science,* attained by a succession of stages of mental action, each above the other. First, there are observation and experiment; next, reflection; then, speculative thinking. These are each discussed in their turn with the same clearness which we have noted in the other disquisitions. The following is the author's summary:

"The process of reason, then, may be described, in conclusion, as a perpetual progress from the real to the ideal, and from the ideal back to the real; at each step becoming more replete with higher thoughts of truth and existence. This progress takes its start from the ordinary intimations of the senses, and tries to find out the general laws which they tacitly involve. It now becomes a matter of doubt whether we do not attribute more to the outward reality than exists there—whether *thought* does not go beyond the corresponding *being.* In proportion as the reason pursues this train of investigation, it falls into one degree of scepticism after another, until a doubt is thrown over the entire reality of human knowledge. Here, however, a regressive principle soon sets in. The reason takes its stand, at length, upon the validity of consciousness. The facts of our internal life, it sees, cannot at all events be denied; so that even if we have no confidence in the reality of the universe, we must at least admit the reality of a series of impressions and ideas corresponding to it."—p. 253.

The reason, however, cannot stop there, but goes on to the conviction, that although there be no matter, in the ordinary sense of the word, there must be a system of *forces,* in the universe—as an existence standing parallel with the world of thought, and participating in the same laws.

"But how can such finite existence be possible? Clearly in the same way as finite thought is possible, namely, as an emanation at once from the infinite being and the infinite mind. Here, then, reason returns virtually to the same realistic point from which it started. It holds once more the validity of the senses, the actuality of the world, the reality of all its phenomena; only it holds them in a higher form, and views them from a loftier point of view. In place of making them the final barrier, on which our powers of thought stumble and break, it sees them all as the unfolding of the infinite essence itself, and reads in them, as exemplars, the laws of *eternal reason, beneficence, and love.*"—p. 254.

The "Methods of Verification," which occupy the last chapter, may be considered as practical illustrations of Mr. Morell's system. All notice of these we must omit, and it therefore only remains for us to express our opinion on that system. It is in every sense of the word eclectic; it is the leading truths of several systems amalgamated into one. As such, it constitutes a key to modern German Philosophy. It is also in all important points a true system. It is founded upon experience and induction; it combines a large number of hitherto heterogeneous phenomena; it links closely the vital to the mental. But Mr. Morell gives only *half* his own system. Insisting upon the unity of soul and body, he takes the soul only into consideration; and when, therefore, we bring the system to the practical test—namely, its bearing on *art,* whether education, legislation, or psychiatrics, we find that the corporeal relations are still to be worked out. In short, although a dual unity in theory, one side only has been developed. To complete his outline, therefore, Mr. Morell has to place neurology and biology parallel with the views we have discussed. Still, as a *system* of psychology, it is, we believe, better adapted to art than any hitherto promulgated.

---

